# Available nitrogen is the key factor influencing soil microbial functional gene diversity in tropical rainforest

**DOI:** 10.1186/s12866-015-0491-8

**Published:** 2015-08-20

**Authors:** Jing Cong, Xueduan Liu, Hui Lu, Han Xu, Yide Li, Ye Deng, Diqiang Li, Yuguang Zhang

**Affiliations:** School of Minerals Processing and Bioengineering, Central South University, Changsha, 410083 China; Institute of Forestry Ecology, Environment and Protection, and the Key Laboratory of Forest Ecology and Environment of State Forestry Administration, Chinese Academy of Forestry, Beijing, 100091 China; Institute of Tropical Forestry, Chinese Academy of Forestry, Guangzhou, 510520 China; Research Center for Eco-Environmental Science, Chinese Academy of Sciences, Beijing, 100085 China

**Keywords:** Tropical rainforest, GeoChip, Microbial functional gene diversity, Microbial metabolic potential, Available nitrogen

## Abstract

**Background:**

Tropical rainforests cover over 50 % of all known plant and animal species and provide a variety of key resources and ecosystem services to humans, largely mediated by metabolic activities of soil microbial communities. A deep analysis of soil microbial communities and their roles in ecological processes would improve our understanding on biogeochemical elemental cycles. However, soil microbial functional gene diversity in tropical rainforests and causative factors remain unclear. GeoChip, contained almost all of the key functional genes related to biogeochemical cycles, could be used as a specific and sensitive tool for studying microbial gene diversity and metabolic potential. In this study, soil microbial functional gene diversity in tropical rainforest was analyzed by using GeoChip technology.

**Results:**

Gene categories detected in the tropical rainforest soils were related to different biogeochemical processes, such as carbon (C), nitrogen (N) and phosphorus (P) cycling. The relative abundance of genes related to C and P cycling detected mostly derived from the cultured bacteria. C degradation gene categories for substrates ranging from labile C to recalcitrant C were all detected, and gene abundances involved in many recalcitrant C degradation gene categories were significantly (*P* < 0.05) different among three sampling sites. The relative abundance of genes related to N cycling detected was significantly (*P* < 0.05) different, mostly derived from the uncultured bacteria. The gene categories related to ammonification had a high relative abundance. Both canonical correspondence analysis and multivariate regression tree analysis showed that soil available N was the most correlated with soil microbial functional gene structure.

**Conclusions:**

Overall high microbial functional gene diversity and different soil microbial metabolic potential for different biogeochemical processes were considered to exist in tropical rainforest. Soil available N could be the key factor in shaping the soil microbial functional gene structure and metabolic potential.

**Electronic supplementary material:**

The online version of this article (doi:10.1186/s12866-015-0491-8) contains supplementary material, which is available to authorized users.

## Background

Tropical rainforests account for only 7 % of the Earth’s land surface, yet they cover over 50 % of all known plant and animal species and provide a variety of key resources and ecosystem services to humans, including food, drinking water, timber and medicines [1–3]. Soil microbial communities are expected to be particularly complex under tropical rainforests [4]. A deep analysis of soil microbial communities and their roles in ecological processes would improve our understanding of biogeochemical elemental cycles in the tropical rainforests [5].

Recent years, there are many reports about microbial community structure based on 16S rRNA sequences or single functional groups in different environments [6, 7]. In the Amazon and other tropical rainforest environments, some studies of soil microbial community composition were mainly examined based on the microbial phylogenetic diversity [8–10]. However, integrated understanding of environmental microbial biogeographic patterns and their ecosystem function is difficult [11]. Therefore, it is needed to explore the microbial functional gene diversity and metabolic potential to detect environmental microbial metabolic processes [7]. GeoChip 5.0, containing almost all of the key functional genes related to biogeochemical cycles, can be used as a specific and sensitive tool for studying microbial gene diversity and metabolic potential [7, 12–14]. Correlations between environmental microbial communities and ecosystem processes have successfully been used in different ecosystems [7, 12–14].

Nitrogen concentration has an important effect on soil microbial community structure and function with further consequences for ecosystem processes [15, 16]. In the last several decades, human activities of deforestation, fossil fuel and fertilizer uses have affected the nitrogen (N) cycling and changed N deposition rate [17, 18], resulting in increased emissions of N at least fourfold over the last century [19]. Elevated atmospheric N deposition is generally considered to significantly alter species composition, nutrient imbalance, nitrate leaching, loss of biodiversity [20]. A number of experiments have been performed to investigate effects of increased input of N on soil microbial community of tropical forests. These studies showed that N enrichment would alter soil microbial community composition and provide feedbacks on soil C pools [19, 20]. However, the microbial ecological process related to N cycling and the key controlling factors in tropical rainforest are unclear. Therefore, it is necessary to discuss microbial functional gene diversity, especially involved in N cycling, and corresponding influencing factors.

Jianfengling Forest Area (JFA) (18°23'–18°52'N, 108°36'–109°05'E) covering 600 km^2^ [21], is situated in southwestern Hainan Island, which is a global biodiversity hotspot area [22], and is one of a few areas in China where primary tropical rainforest is preserved. The forest area is heavily affected by N deposition, with over 25 kg N ha^-1^ yr1^-1^ deposited on Hainan Island from 1990 to 2003 [23]. In this study, we selected three sampling sites in the primary tropical rainforest in Jianfengling and analyzed the soil microbial functional gene diversity and metabolic potential using GeoChip 5.0. The aims were to determine (i) soil microbial functional gene diversity and metabolic potential in primary tropical rainforest soils; (ii) major environmental factors in governing the soil microbial functional gene diversity.

## Results

### Plant communities and soil properties

According to (Additional file 1: Table S1 and S2), plant dominant species and plant species diversity were distinctly different among three sampling sites. The JFL-3 and JFL-1 had the highest and lowest Shannon-Weaver index, respectively. The PCA showed that three sampling sites were well separated from each other (Additional file 2: Figure S1), indicated that they had distinct plant community structure. Soil pH was acidity (pH < 5.0), and soil moisture was over 30 % in three sampling sites. For the soil nutrient properties, these sites were also different. The JFL-3 had the highest contents in soil total nitrogen and available nitrogen. Therefore, plant communities and soil properties had differences among three sampling sites.

### Microbial functional gene diversity in tropical rainforest soil

**Fig. 1:**
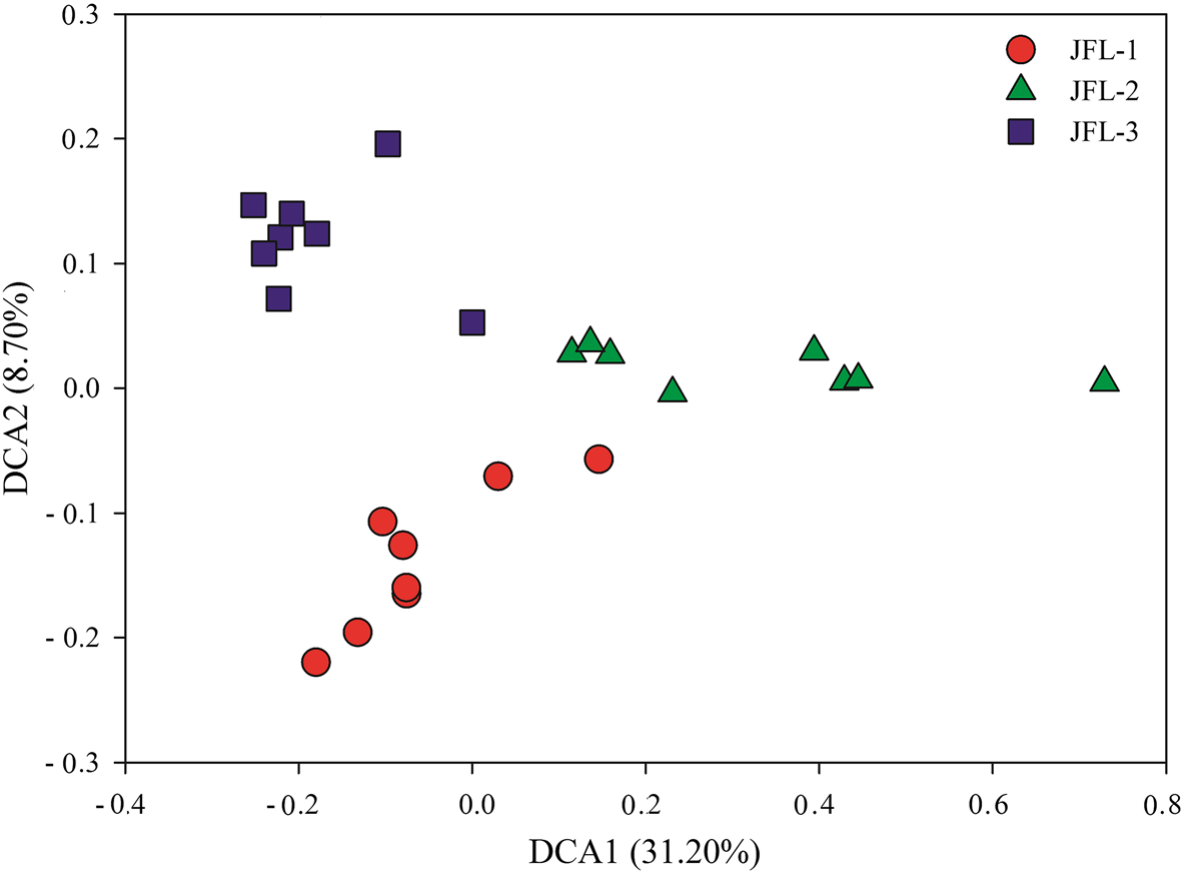
Detrended correspondence analysis (DCA) of soil microbial community based on functional gene data. The DCA was analyzed based on the relative signal intensity of functional genes (*n* = 24)

To understand soil microbial functional gene diversity, the detected number of genes, alpha diversity index (Shannon and Simpson index) and normalized signal intensity of microbial functional gene families were analyzed. The total number of genes detected was 27,887, ranged from 22,902 to 27,363 in the three sampling sites (Table 1), which accounted for 45.90 %, 39.83 % and 47.58 % of the total gene probes of GeoChip 5.0 in JFL-1, JFL-2 and JFL-3, respectively. The mean number of detected genes and alpha diversity index were the lowest in JFL-2 (19,469 and 9.85, respectively). The DCA of all detected genes showed that soil microbial functional communities were well separated from three sampling sites (Fig. 1), indicated that distinct microbial functional gene structure existed in the three sampling sites.

**Table 1:** Gene number and diversity indices for GeoChip data in three sampling sites

Parameter	JFL-1	JFL-2	JFL-3
No. of detected genes	26392	22902	27363
Mean No. of detected genes^a^	24497 ± 1355a	19469 ± 3347b	25625 ± 954a
Shannon index^a^	10.09 ± 0.06a	9.85 ± 0.19b	10.14 ± 0.04a
Simpson index^a^	23930 ± 1286a	19110 ± 3228b	25037 ± 932a

^a^The data is the mean value and standard error for eight plots. The same lowercase letters within the same row in the footnote mean the difference was not significant, whereas the difference was significant (*P* < 0.05)

Of the 393 functional gene categories based on GeoChip 5.0, 89.06 % was detected. The detected functional gene categories were related to different biogeochemical processes, such as C cycling, N cycling, S cycling, P cycling, organic remediation, metal homeostasis and virulence (Additional file 2: Figure S2). These functional gene categories were distinctly different among these sampling sites. JFL-2 had significantly (*P* < 0.05) higher functional gene abundances of C cycling, P cycling and S cycling genes than these in JFL-1 and JFL-3. For function gene abundances involved in N cycling, metal Homeostasis, organic remediation and virulence, JFL-2 was significantly (*P* < 0.05) lower than in JFL-1 and JFL-3. To better understand microbial functional gene diversity and metabolic potential, key gene categories involved in C, N and P cycling were selected and analyzed.

#### Microbial functional genes involved in C cycling

**Fig. 2:**
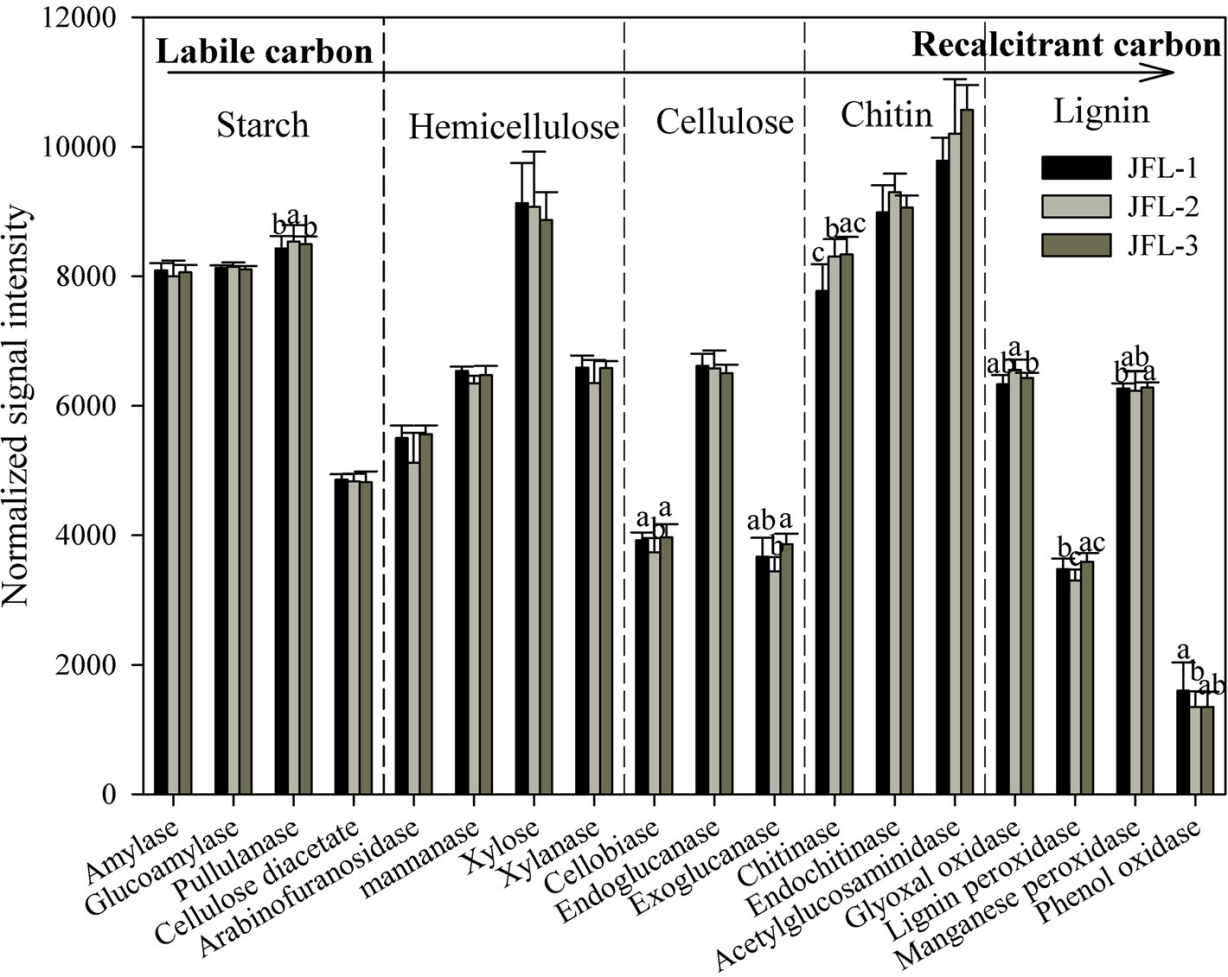
The normalized signal intensity of the detected key genes involved in carbon degradation. The complexity of carbon is presented in order from labile to recalcitrant. The signal intensity for each functional gene category is the average of the total signal intensity from all the replicates (*n* = 8). All data are presented as mean ± SE (error bars). Different letters indicated statistical differences at a *P* value of < 0.05 among sampling sites by one-way ANOVA

Key functional gene categories involved in carbon degradation, carbon fixation, methane production and methane oxidation were detected in all sampling sites. A total of 8,578 genes involved in carbon degradation were detected in these sampling sites, and most of the functional gene (95.44 %) involved in carbon degradation was derived from cultured bacteria. Among the three sampling sites, the functional genes related to recalcitrant carbon degradation encoding cellobiase, exoglucanase, chitinase, glyoxal oxidase, lignin peroxidase, manganese peroxidase and phenol oxidase were significantly (*P* < 0.05) different (Fig. 2). For example, the abundance of genes encoding cellobiase was significantly lower (*P* < 0.05) in JFL-2 compared to the other two sampling sites. However, there were no significant differences for functional gene abundance related to labile carbon (starch and hemicellulose) degradation among the three sampling sites, except for labile carbon degradation gene *pullulanase*, which was significantly (*P* < 0.05) higher in JFL-2 than in JFL-1 and JFL-3.

A total of 3,131 and 228 genes involved in carbon fixation and methane cycling were detected in these sampling sites, respectively. The key genes (*FBPase*, *rubisco*, *CODH*, and *FTHFS*) involved in carbon fixation were detected in all sampling sites and most of the detected genes (92.14 %) were derived from cultured bacteria. The abundance of genes involved in the Calvin cycle was significantly (*P* < 0.05) lower in JFL-2 than in JFL-1 and JFL-3 (Additional file 2: Figure S3). For the reductive acetyl-CoA pathway, the gene abundance of *CODH* was significantly higher in JFL-2 than in JFL-1 and JFL-3, yet *FTHFS* was significantly lower in J FL-2 than in JFL-1 and JFL-3 (Additional file 2: Figure S3). For methane cycling, 78.51 % of the detected genes were derived from uncultured bacteria. Genes (*mcrA*, *mmoX* and *pmoA*) involved in methane production and oxidation were detected in all sampling sites (Additional file 2: Figure S4). The abundance of *mcrA* genes involved in methane production in JFL-2 was significantly (*P* < 0.05) higher than in JFL-1 and JFL-3, and the abundance of *pmoA* genes involved in methane oxidation in JFL-2 was significantly lower (*P* < 0.05) compared to the other two sampling sites.

These results showed that all of the metabolic processes soil bacteria mediated of related to carbon degradation, carbon fixation and methane cycle existed in these tropical rainforest soils, and some key functional gene abundances were significantly different (*P* < 0.05), which could lead to the differences of soil microbial metabolic potential among these sampling sites.

#### Microbial functional genes involved in N cycling

**Fig. 3:**
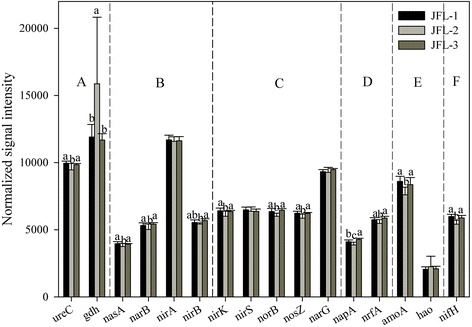
The normalized signal intensity of the detected key genes involved in nitrogen cycling. The signal intensity for each functional gene category is the average of the total signal intensity from all the replicates (*n* = 8). (**a**). Ammonification, including *gdh* for glutamate dehydrogenase and *ureC* encoding urease; (**b**). Assimilatory N reduction, including *nasA* encoding nitrate reductase, *narB*, *nirA* and *nirB* encoding dissimilatory nitrite reductase; (**c**). Denitrification, including *narG* for nitrate reductase, *nirS* and *nirK* for nitrite reductase, *norB* for nitric oxide reductase, and *nosZ* for nitrate reductase; (**d**). Dissimilatory N reduction, including *napA* for nitrate reductase, and *nrfA* for c-type cytochrome nitrite reductase; (**e**). Nitrification, including *amoA* encoding ammonia monooxygenase, *hao* for hydroxylamine oxidoreductase; (**f**). N fixation, including *nifH* encoding nitrogenase. All data are presented as mean ± SE (error bars). Different letters indicated statistical differences at a *P* value of < 0.05 among sampling sites by one-way ANOVA

A total of 3,624 genes involved in N cycling were detected in these sampling sites, including ammonification, assimilatory N reduction, denitrification, nitrification, dissimilatory N reduction and N fixation, and 60.13 % of these genes were derived from uncultured bacteria. Gene abundances related to assimilatory N reduction, denitrification, dissimilatory N reduction, nitrification, and N fixation were significantly lower in JFL-2 than these in JFL-1 and JFL-3 (*P* < 0.05; Fig. 3). These results showed that almost all the metabolic processes related to N cycling were present in these tropical rainforest soils, while the metabolic potential could be discrepant among three sampling sites in the tropical rainforest soils.

#### Microbial functional genes involved in P cycling

Total of 1,208 functional genes involved in P cycling were detected in these sampling sites, including polyphosphate degradation (polyphosphatase gene, *ppx*), polyphosphate synthesis (polyphosphate kinase gene, *ppk*) and phytic acid hydrolysis (phytase gene, *phy*), and most of the detected genes (92.47 %) were derived from cultured bacteria. The abundances of *ppx* and *phy* genes in JFL-2 were significantly higher (*P* < 0.05) than in JFL-1 and JFL-3, while the abundance of *ppk* in JFL-2 was significantly lower (*P* < 0.05) than in JFL-1 and JFL-3 (Additional file 2: Figure S5). These results showed that all the metabolic processes related to P cycling were presented in these tropical rainforest soils, and metabolic potential for P cycling might be different in these three sampling sites.

### Relationship between soil microbial functional gene structure and environmental variables

**Fig. 4:**
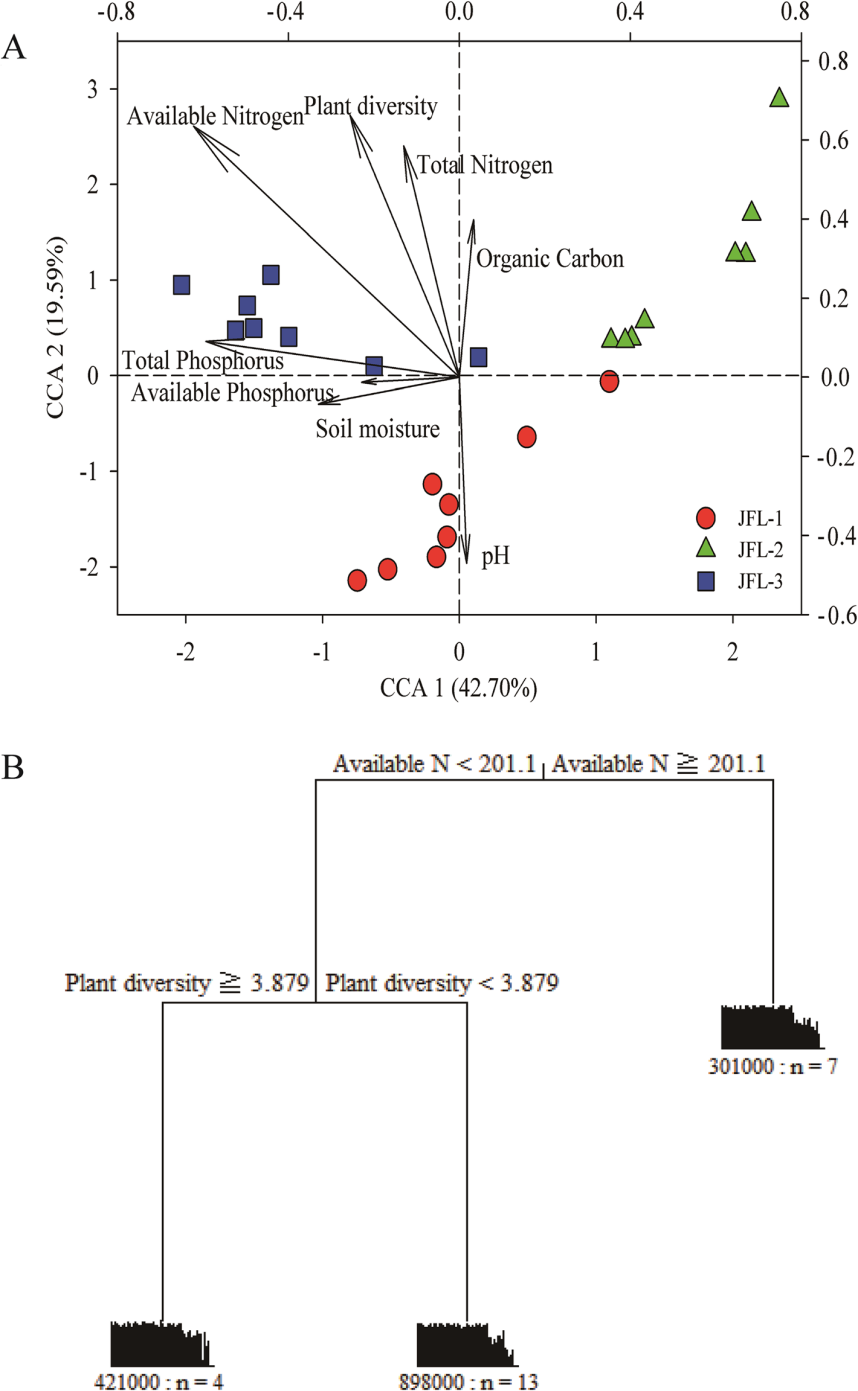
The linkage of soil microbial communities and environmental variables. (**a**). Canonical correspondence analysis (CCA) of soil microbial communities and environmental variables based on GeoChip data, (**b**) Multivariate regression tree (MRT) of soil microbial communities associated with three sampling sites. The units of available N and plant diversity were mg/kg and 1; n, 421000 (898000 and 301000) represented the sample number and relative error, respectively

Canonical correspondence analysis (CCA) was used to analyze the relationship between the microbial functional gene structure and the major environmental variables (Fig. 4), resulting in a significant model at a confidence level of *P* = 0.043. The first axis was the most negatively and significantly correlated with soil available N, followed by total phosphorus. The second axis was the most positively and significantly correlated with plant diversity, followed by total nitrogen (Fig. 4a). Therefore, N, especially soil available N, was the most correlated with soil microbial communities at the functional gene level.

Multivariate regression tree (MRT) analysis is well suitable for complex ecological datasets with high-order interactions in a visualized tree [24]. For the MRT analysis, dissimilarity was used to split data into two groups based on one of the environmental variables. Each split was described graphically as a branch in a tree. Each branch was labeled with response environmental variable that was placed in that branch. We compared the results of MRT using the whole GeoChip data and environmental variables (Fig. 4b). In this tree, soil microbial communities at the functional gene level were first split by soil available N, followed by plant species diversity. According to the relationship between environmental factors and microbial functional gene structure, soil available N could be the major factors in shaping the microbial functional structure in these sampling sites, followed by plant species diversity, similar to the CCA result.

## Discussion

GeoChip-based data provided a lot of information on different biogeochemical processes, which contributed to explore soil microbes potentially effects on soil enzyme activities or components. In this study, we analyzed soil microbial functional gene structure using GeoChip 5.0, and detected microbial functional gene categories, which could represent the *in situ* metabolic potential to some degree [7]. Soil carbon cycling is one of the most important and complex processes in biogeochemical cycles [25]. In our study, the gene number of functional genes related to carbon cycling accounted for over 40 % of all genes detected, suggested that these tropical rainforest soils might have a strong metabolic potential for carbon cycling.

Humid tropical forest soils have the fastest degradation rates of plant litter [26], conducted by soil microorganisms with highly efficient carbon degrading ability. For example, cellulose enzyme activity related to cellulose gene variants [27, 28] and oxidizable organic carbon was significantly linked to the relative abundance of genes involved in cellulose, hemicellulose and starch degradation [7]. Carbon degradation gene categories for substrates ranging from labile carbon (starch and hemicellulose) to recalcitrant carbon (cellulose, chitin and lignin) were all detected in three sampling sites. The relative abundance of most functional genes involved in recalcitrant carbon degradation (e.g., cellulose, chitin and lignin) was distinctly different among the three sampling sites, which indicated that the metabolic potential of degrading recalcitrant carbon for soil microbial communities was discrepant from three sampling sites in tropical rainforest. In addition, the relative abundance of genes related to active carbon degradation was not significantly different among the sampling sites, except for *pullulanase* gene, which indicated that soil microbes related to labile carbon degradation could execute similar carbon substrates. Therefore, tropical rainforest soils may contain a series of complex, adaptable microbial metabolic communities involved in degradation of different substrates.

P cycling played a key role in changing the species composition, diversity and productivity in tropical rainforests. In this study, we detected a high relative abundance of *phy*, *ppk* and *ppx* genes (Additional file 2: Figure S5). Microbial mineralization of phytate, which is the most abundant compound containing organic P in soil, is a key process for recycling P by phytases in the biosphere [29]. At present, four classes of phytases have been defined in terrestrial organisms: histidine acid phosphatase, cysteine phytase, purple acid phosphatase and β–propeller phytase [29, 30]. *ppk* encodes polyphosphate kinase, which could be used to reflect the potential for biological P removal [31]. *ppx* encodes exopolyphosphatase, catalyzing the polyphosphate transfer to phosphate. The tropical rainforests are considered to be P limited [32], since elevated atmospheric N deposition may further lead to P limitation [20]. Experimental P additions in tropical rainforests have revealed that microbial utilization for the labile fractions of soil organic C could be restricted or controlled by soil P [33]. Moreover, P-induced variation in microbial communities could cause corresponding shifts in the functional and metabolic potentials of the communities, resulting in a change in decomposition rates [15]. The CCA result also indicated that soil total phosphorus played a key role in soil microbial communities (Fig. 4a). In general, a highly efficient bacterial community structure and metabolic potential was formed to adapt to P limitation in the tropical rainforest.

Soil microbes are the major drivers of soil N cycling, including N fixation, nitrification, denitrification, ammonification, assimilatory N reduction and dissimilatory N reduction [34]. In our study, we found a high metabolic potential for nitrate reduction, especially a high relative abundance of the *narG* gene, which could be caused by the high rate of N deposition in forest soils [20]. Some tropical forests were observed to be N saturated and exhibited a high nitrate leaching rate in the region of high N deposition region [35], particularly the old-growth forest [36]. Nitrogen from ammonium (NH_4_^ +^) is the dominant N form in bulk deposition, however, the increasing rate for N deposition is the highest from nitrate (NO_3_^ -^) [36]. To decrease NO_3_^ -^ concentrations in soil, a strong metabolic potential for denitrification is needed. High soil N_2_O fluxes were observed (mean N_2_O emission rate of 2.21 kg N-N_2_O ha^-1^ yr^-1^ in the wet season) in the JFL [37], which were significantly higher than temperate spruce forest in Germany, tropical rainforests in Australia and Indonesia, and un-grazed semi-arid steppe in China [38, 39]. Therefore, microbial communities from the tropical rainforest had a highly efficient metabolic potential in satisfying the soil N cycling.

In this study, we detected almost all the functional gene categories involved in N cycling based on GeoChip 5.0. There were distinct differences in the relative abundance of different functional gene categories, indicated that metabolic potentials for different processes in N cycling were distinctly different, probably caused by N limiting factors in the ecosystem. N is the primary factor of limiting plant growth in tropical rainforests, and strong competition has been observed between plants and microorganisms [40]. Nitrogen concentration played a key role in soil microbial community structure and function with further consequences for ecosystem processes [15, 16]. N additions could directly restrict microbial growth [41], relative abundance [42] and microbial functional diversity [43]. Krashevska et al. found that the soil microbial biomass and community structure were strongly altered in response to even moderate changes in N inputs in the soils of tropical rainforests, indicating that the potential was changed in causing key ramifications of the whole ecosystem including plant growth and litter decomposition through nutrient additions [19]. Many microbial enzyme activities were changed by N condition [44]. For example, cellulase activity of decaying oak litter was stimulated by N addition, but lignin–degrading activity was significantly decreased [45]. Also, N saturation could also lower soil pH, resulting in leaching of calcium and magnesium and mobilization aluminum, and then experiencing aluminum toxicity [46]. In our study, both CCA and MRT analyses indicated that soil available nitrogen was the most important factors in influencing soil microbial community structure at the functional gene level.

In addition, microbial communities mediated soil key processes involved in carbon and nitrogen cycling, which potentially represented a mechanistic relationship between plant species diversity and ecosystem function. The availability of growth-limiting resources, such as organic compounds in dead leaves and roots (i.e., detritus) that can generate cellular energy [47], influences the composition of microbial communities [48]. Due to different biochemical composition and changes in plant diversity, plant species changes the production and range of organic compounds in detritus that influence the composition and function of heterotrophic microbial communities. Zak DR, et al. conducted a long-term field manipulation to determine that the biomass, respiration and abundance of soil microbial communities significantly increased with higher plan species diversity [49]. The PCA indicated that plant communities were distinct among three sampling sites (Additional file 2: Figure S1). Therefore, plant communities could have an important control in microbial community structure, verified by CCA and MRT analyses.

## Conclusions

In summary, we analyzed the soil microbial functional gene diversity and metabolic potential in the tropical rainforests of JFL by GeoChip technology. The metabolic processes soil bacteria mediated of related to C, N, P cycling existed in these tropical rainforest soils, and some key functional gene abundances were significantly different (*P* < 0.05), which could lead to the differences of soil microbial metabolic potential among these sampling sites. A high relative abundance of genes related to ammonification, denitrification and N fixation were found in these soils. Both CCA and MRT showed that soil microbial functional gene structure was mainly influenced by soil available N and plant diversity. These results indicated the metabolic potential for microbial communities could acclimatize to acid tropical rainforest soils.

## Methods

### Site and sampling

The study sites were located on JFA (Additional file 2: Figure S6) and botanically diverse with over 2,800 plant species [46]. The main forest types include tropical semi-deciduous monsoon forest, tropical evergreen monsoon forest, tropical montane rainforest, and mossy forest on the mountain peak. The climate is characterized by tropical monsoons with a wet season from May to October and a dry season from November to April. The mean annual temperature is 24.5 °C, with an annual rainfall of 1,600–2,600 mm [50].

In the JFA, we selected three sampling sites (JFL-1, JFL-2 and JFL-3) in the tropical rainforest of well-protected primary forest types. In three sampling sites, there existed different plant dominant species, which were *Gironniera girosuba*, *Blastus blascoch* and *Pinanga pinabavi* in JFL-1, *Gironniera girosuba*, *Cryptocarya crypchine* and *Prismatomeris pristetr* in JFL-2, Neolitsea *Gironniera girosuba*, *Cryptocarya crypchine* and *Neolelli* in JFL-3, respectively (Additional file 1: Table S1). The mean altitude was 872 m, 945 m, and 992 m in JFL-1, JFL-2 and JFL-3, respectively.

These soil samples were collected in March 2012. In each sampling site, eight plots of 20 m × 20 m were carried out with a plot interval between adjacent sampling plots, total of 24 plots sampled (Additional file 2: Figure S6). Soil samples were randomly taken from each plot at the depth of 10 cm by multiple sampling methods, and ten to fifteen points in each plot were collected, and mixed as a plot soil sample. Roots and stones were removed from samples. Soil samples were stored at 4 °C for soil physicochemical analysis, and at –80 °C for DNA analysis, respectively.

### Plant diversity and soil nutrient properties

All trees with diameter at breast height (1.3 m; DBH) of ≥ 1.0 cm were surveyed to profile plant communities. The plant Shannon-Weaver index of each plot was calculated according to the surveyed plant data (Additional file 1: Table S2). Soil moisture (MO) was measured by gravimetrical methods. Soil pH was measured with a pH meter by soil water (1:2.5 mass: volume soil and water suspension). Soil total nitrogen (N) content, total phosphorus (P) content, available nitrogen (N) content, total organic carbon (C) content and pH were analyzed as previously described [51].

### Microbial DNA extraction, purification and quantification

As previously described [52], soil microbial DNA was extracted directly from 5 g combined soil by freeze-grinding mechanical lysis. DNA quality and concentration were evaluated using a FLUOstar Optima microplate reader (BMG Labtech, Jena, Germany), based on the ratios of 260 nm/280 nm and 260 nm/230 nm.

### GeoChip hybridization and data analysis

GeoChip was used to analyze soil microbial functional gene diversity and metabolic potential. GeoChip 5.0 contained about 57,504 50-mer oligonucleotide probes, covered 393 gene categories, and involved in microbial (protists, fungi, bacteria and archaea) biogeochemical cycles of carbon, nitrogen, phosphorus, sulfur, etc. (http://ieg.ou.edu/entrance.html). Soil microbial DNA purification, labeling, GeoChip hybridization and signal reading were performed according to previous methods [7, 13]. GeoChip data were further analyzed using the following steps: (i) genes detected in only two out of eight samples from the same sampling site were removed; (ii) signals were normalized by dividing by the mean value of total signal intensity of 24 samples.

### Statistical analysis

The alpha diversity of soil microbial communities was conducted by the Shannon-Weaver index and Simpson’s index based on the GeoChip data. The normalized signal intensity of each functional gene family was used as the gene relative abundance [12]. Statistical differences were analyzed by a one-way analysis of variance (ANOVA) at a significance level of *P* < 0.05. Principal component analysis (PCA) was used to evaluate the differences of plant communities among sampling sites. Detrended correspondence analysis (DCA) was used to evaluate the differences of soil microbial communities among sampling sites, carried out by using the ‘vegan’ package. Canonical correspondence analysis (CCA) and multivariate regression tree (MRT) were used to explain the relationship of soil microbial community structure and environmental variables in a visualized tree, carried out by using the ‘vegan’ and ‘mvpart’ package, respectively. All the data analyses were performed in R (v. 2.13.1).

### Availability

The microarray data discussed in this manuscript have been deposited in NCBI's Gene Expression Omnibus and are accessible through GEO Series accession number GSE6917 (http://www.ncbi.nlm.nih.gov/geo/query/acc.cgi?acc=GSE69171).

## Additional files

Additional file 1: Table S1. The general characters of three sampling sites in the tropical rainforest. **Table S2.** The environmental factors of three sampling sites in the tropical rainforest. (DOC 40 kb) Additional file 2: Figure S1. The principal component analysis of plant community structure in three sampling sites. **Figure S2.** The normalized signal intensity of detected genes from different gene categories. **Figure S3.** The normalized signal intensity of detected key genes involved in carbon fixation. **Figure S4.** The normalized signal intensity of detected key genes involved in methane cycle. **Figure S5.** The normalized signal intensity of the detected key genes involved in phosphorus cycle. **Figure S6.** Study sites in Jianfengling Forest Area. (DOCX 1154 kb)
