# Supplementary material for: Available nitrogen is the key factor influencing soil microbial functional gene diversity in tropical rainforest
Source: BMC Microbiol. 2015 Aug 20;15:167. doi: 10.1186/s12866-015-0491-8 (PMC4546036; doi:10.1186/s12866-015-0491-8)
Supplement: Additional file 1: Table S1. — The general characters of three sampling sites in the tropical rainforest. Table S2. The environmental factors of three sampling sites in the tropical rainforest. (DOC 40 kb) [file 12866_2015_491_MOESM1_ESM.doc]

Table S1 The general characters of three sampling sites in the tropical rainforest

| Study site | Quadrat number | Dominant species | Altitude (m) | Slope (°) | Convexity (m) |
| --- | --- | --- | --- | --- | --- |
| JFL-1 | 8 | *Gironniera girosuba*-*Blastus blascoch*-*Pinanga pinabavi* | 872.05±2.58 | 7.03±5.29 | 0.65±0.83 |
| JFL-2 | 8 | *Gironniera girosuba*-*Cryptocarya crypchine*-*Prismatomeris pristetr* | 944.79±5.89 | 27.28±6.04 | 1.95±1.36 |
| JFL-3 | 8 | *Gironniera girosuba*-*Cryptocarya crypchine*-*Neolitsea Neolelli* | 991.68±13.47 | 26.55±3.00 | 1.06±1.12 |

The data is the mean value and standard error for 8 plots.

Table S2 The environmental factors of three sampling sites in the tropical rainforest

| Environmental factors | JFL-1 | JFL-2 | JFL-3 |
| --- | --- | --- | --- |
| Soil moisture (%) | 34.28±4.67ab | 32.03±2.14b | 36.57±5.31a |
| Soil pH | 4.52±0.08a | 4.36±0.12b | 4.37±0.12b |
| Soil organic carbon (g/kg) | 47.09±4.40a | 55.63±15.88a | 55.47±4.86a |
| Soil total nitrogen (g/kg) | 1.79±0.21b | 2.22±0.47a | 2.35±0.31a |
| Total phosphorus (g/kg) | 0.12±0.01a | 0.12±0.01a | 0.14±0.03a |
| Available nitrogen (mg/kg) | 141.37±12.80c | 175.27±27.49b | 224.45±24.911a |
| Available phosphorus (mg/kg) | 2.72±0.74a | 2.97±0.97a | 3.15±0.95a |
| Plant species number | 74.13±9.03a | 80.75±15.57a | 79.63±11.89a |
| Plant Shannon-Weaver index | 3.73±0.11b | 3.82±0.16ab | 3.95±0.13a |

The data is the mean value and standard error for 8 plots. The same lowercase letters within the same row represent the difference was not significant, whereas the difference was significant (*P* < 0.05).
