# Supplementary material for: Available nitrogen is the key factor influencing soil microbial functional gene diversity in tropical rainforest
Source: BMC Microbiol. 2015 Aug 20;15:167. doi: 10.1186/s12866-015-0491-8 (PMC4546036; doi:10.1186/s12866-015-0491-8)
Supplement: Additional file 2: Figure S1. — The principal component analysis of plant community structure in three sampling sites. Figure S2. The normalized signal intensity of detected genes from different gene categories. Figure S3. The normalized signal intensity of detected key genes involved in carbon fixation. Figure S4. The normalized signal intensity of detected key genes involved in methane cycle. Figure S5. The normalized signal intensity of the detected key genes involved in phosphorus cycle. Figure S6. Study sites in Jianfengling Forest Area. (DOCX 1154 kb) [file 12866_2015_491_MOESM2_ESM.docx]

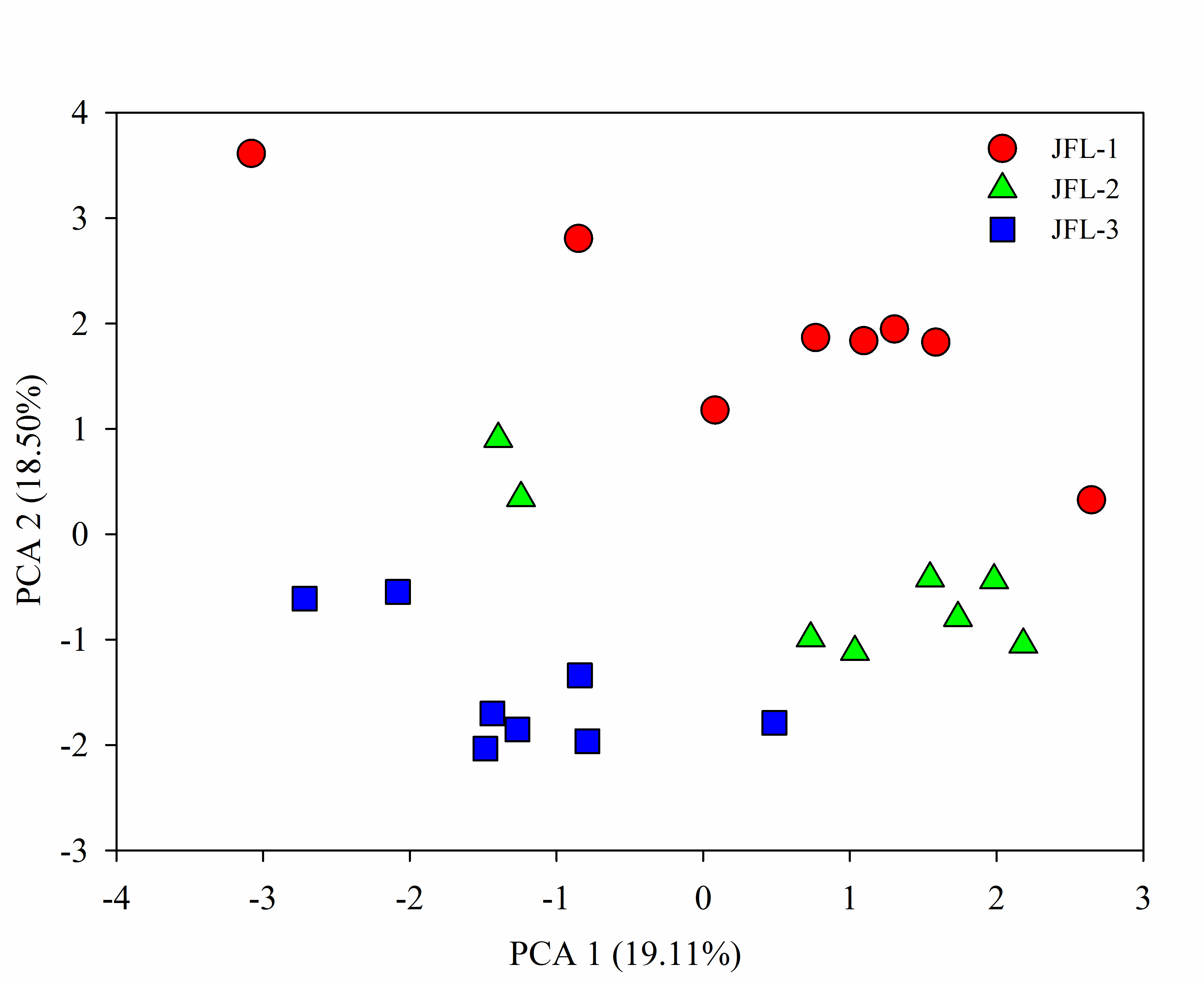


Figure S1 Principal component analysis (PCA) of plant community structure among three sampling sites.


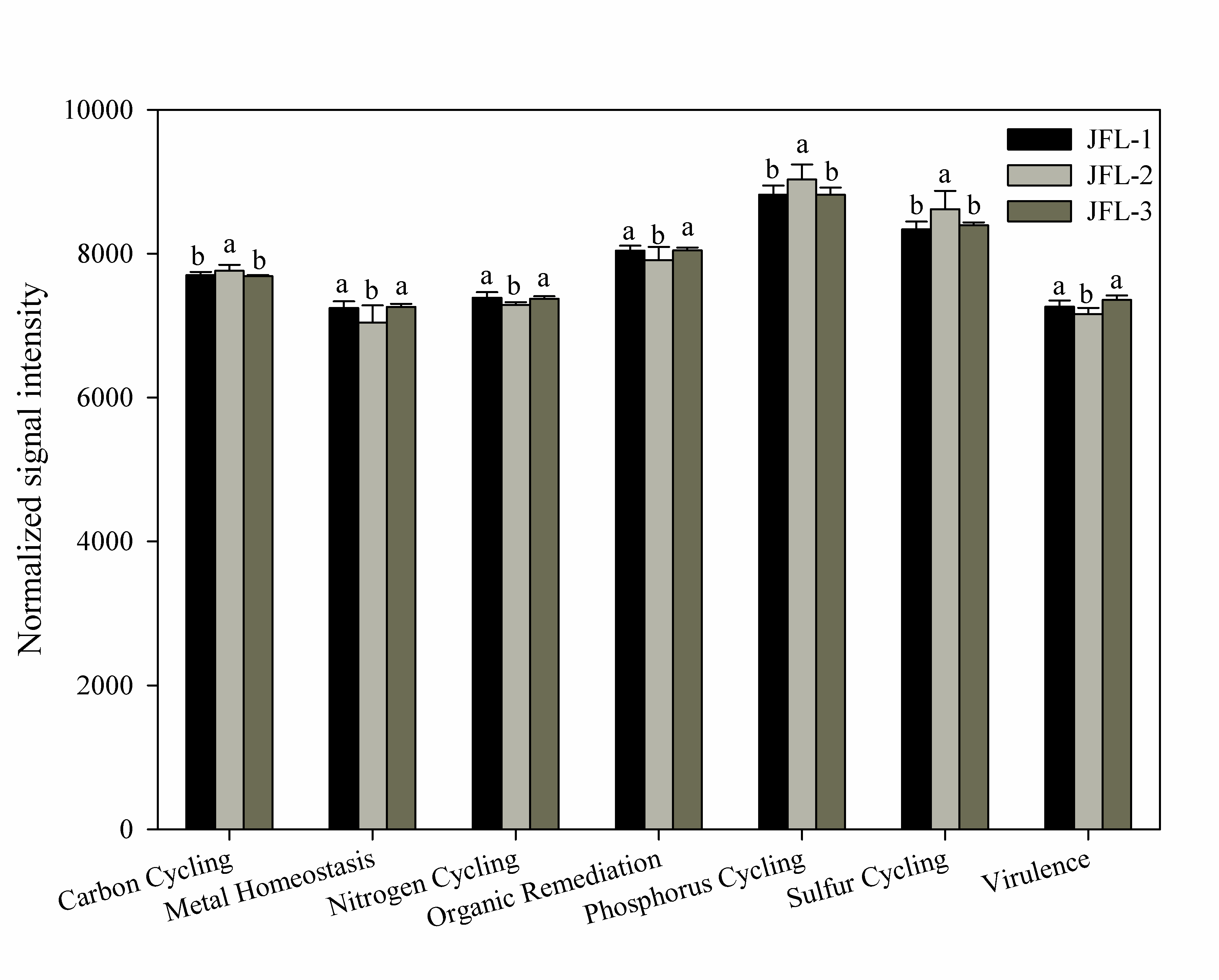


Figure S2 The normalized signal intensity of detected genes from different gene categories. The signal intensity for each functional gene category is the average of the total signal intensity from all the replicates. All data are presented as mean ± SE (n=8).


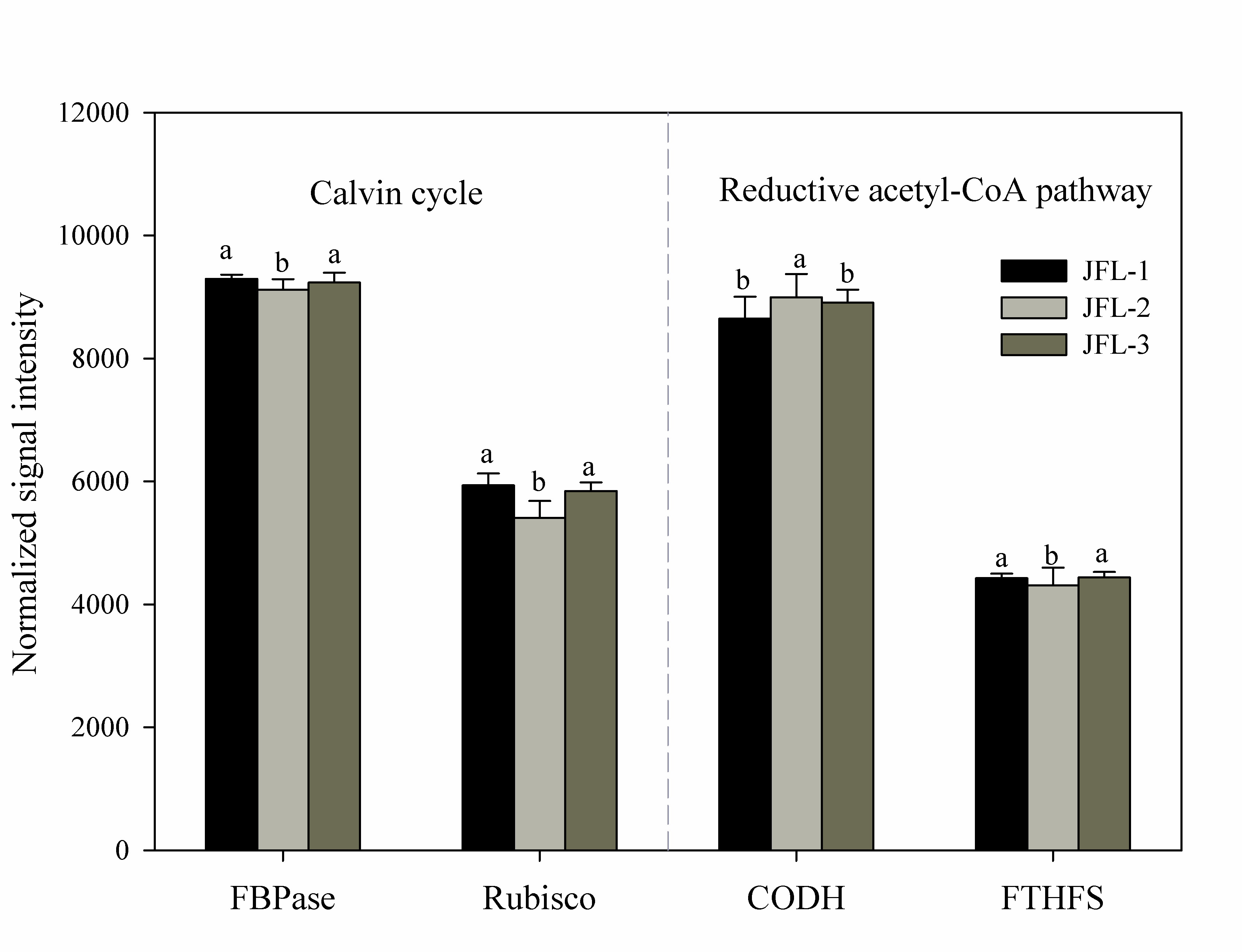


Figure S3 The normalized signal intensity of detected key genes involved in carbon fixation. The signal intensity for each functional gene category is the average of the total signal intensity from all the replicates. *CODH*: carbon monoxide dehydrogenase; *Rubisco*: ribulose-1,5-bisphosphate carboxylase/oxygenase. All data are presented as mean ± SE (n=8). Different letters indicated statistical differences at a *P* value of < 0.05 among sampling sites by one-way ANOVA.


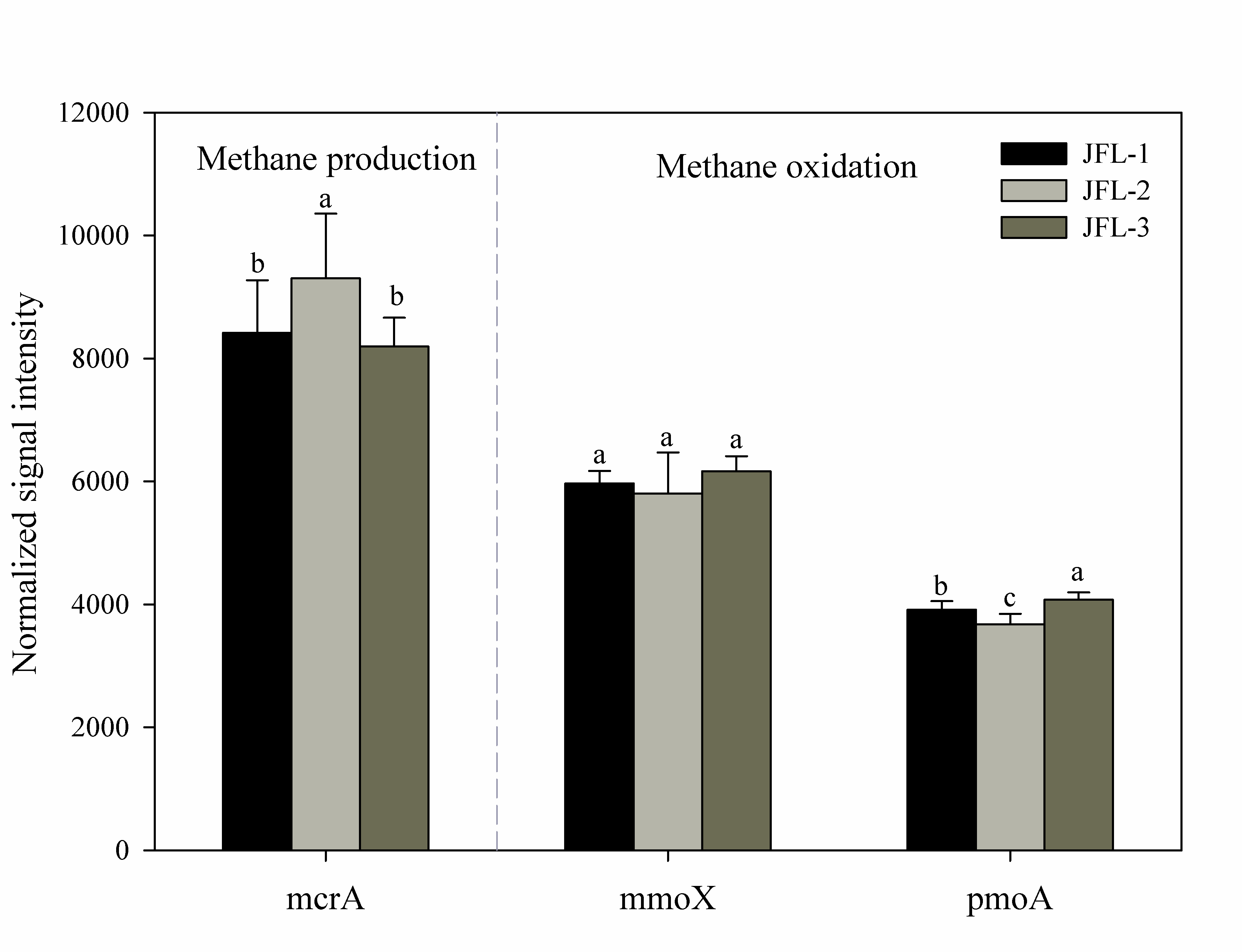


Figure S4 The normalized signal intensity of the detected key genes involved in methane cycle. The signal intensity for each functional gene category is the average of the total signal intensity from all the replicates. *pmoA*: methane monooxygenase; *mcrA*: methyl coenzyme M reductase. All data were presented as mean ± SE (n=8). Different letters indicated statistical differences at a *P* value of < 0.05 among sampling sites by one-way ANOVA.


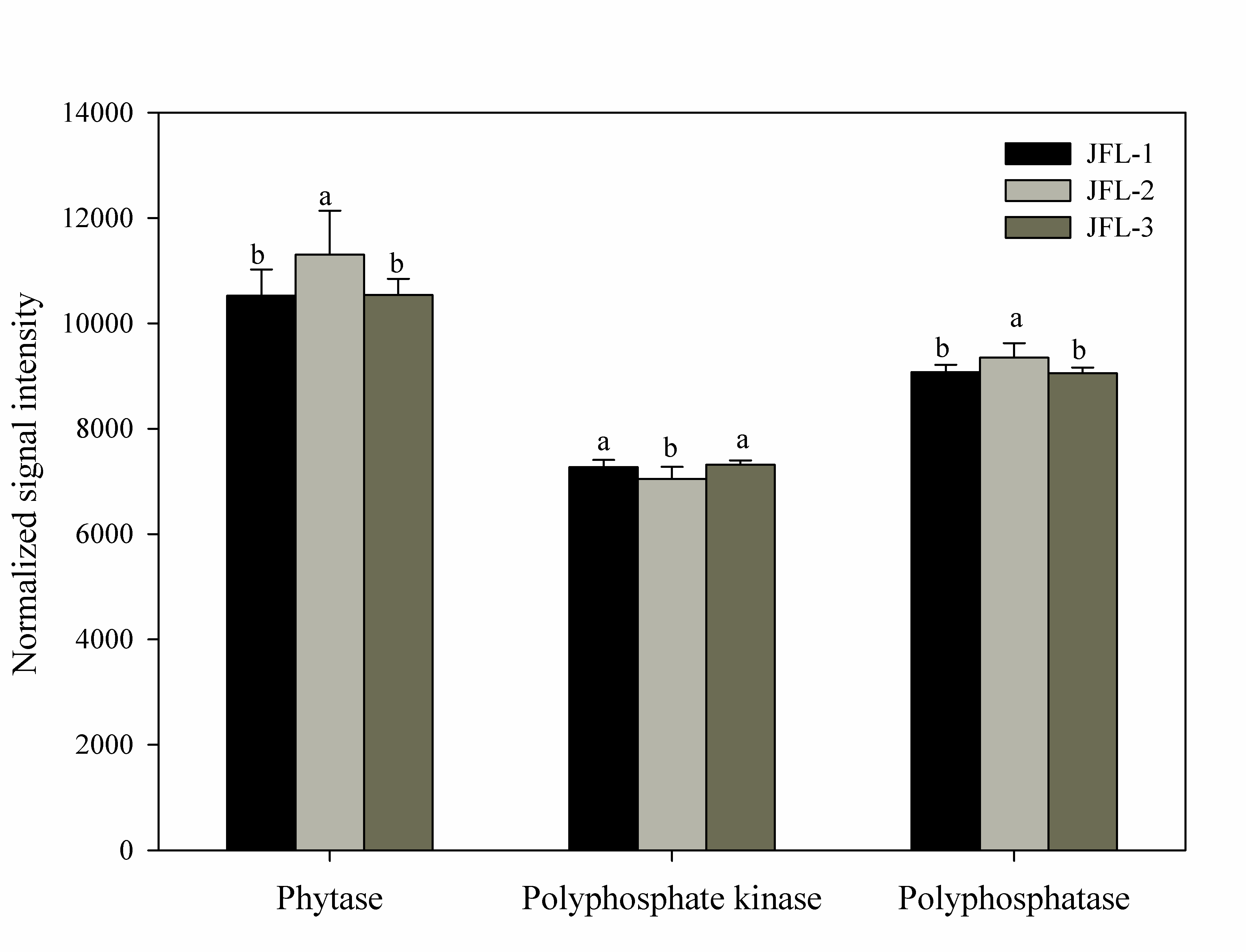


Figure S5 The normalized signal intensity of the detected key genes involved in phosphorus cycle. The signal intensity for each functional gene category is the average of the total signal intensity from all the replicates (n=8). All data are presented as mean ± SE (error bars). Different letters indicated statistical differences at a *P* value of < 0.05 among sampling sites by one-way ANOVA.


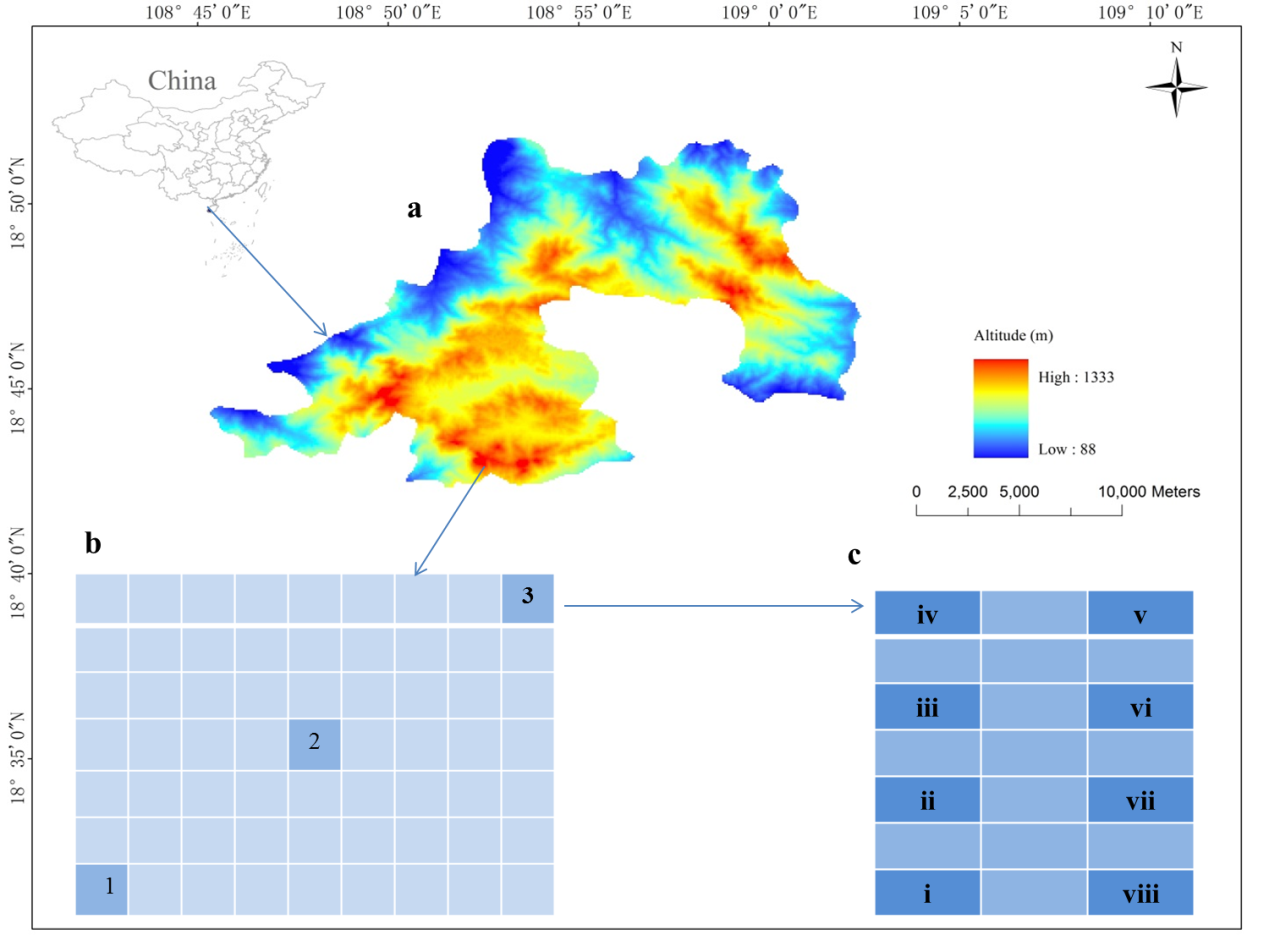


**Figure S6** Study sites in Jianfengling Forest Area. (a) Jianfengling Forest Area (JFA); (b) three sampling sites including JFL-1, JFL-2, and JFL-3, with about a 500 meters distance between each other; (c) In each sampling site, eight quadrats of 20 m × 20 m were carried out with a quadrat interval between adjacent sampling quadrats, total of 24 quadrats sampled.
